# Erector spinae block reduces intraoperative and postoperative opioid consumption in patients undergoing laparoscopic sleeve gastrectomy: A randomized controlled trial

**DOI:** 10.5339/qmj.2024.58

**Published:** 2024-12-26

**Authors:** Anwar ul Huda, Amer Saeed Alshahrani, Mohammad Yasir, Abdulaziz Sawilah, Ahmed Abdulrahman N Alharthi

**Affiliations:** 1 Department of Anesthesia, Security Forces Hospital, Riyadh, Kingdom of Saudi Arabia *Email: hudaanwar90@yahoo.com; 2 Department of Surgery, Security Forces Hospital, Riyadh, Kingdom of Saudi Arabia

**Keywords:** Erector spinae plane block, sleeve gastrectomy, pain score, opioid consumption

## Abstract

**Background:**

Obese patients are at increased risk of postoperative respiratory complications because of sedatives and opioids. The erector spinae block is a novel regional block that has been used in different surgeries. It offers an easier approach and a better safety profile. This study aimed to assess the role of erector spinae plane block (ESPB) in reducing postoperative pain scores and opioid consumption in patients undergoing sleeve gastrostomies.

**Methods:**

Institutional committee approval was obtained for this randomized controlled trial. Inclusion criteria included patients aged between 18 and 65 years with American Society of Anesthesiologists (ASA) scores 1–3 who were scheduled to undergo laparoscopic sleeve gastrectomy under general anesthesia. Simple randomization using sealed opaque envelopes was used to allocate study patients to either of the two groups. The intervention group received erector spinae block using 0.2% ropivacaine just after induction of anesthesia while the control group did not receive a block. Primary outcome variables were pain scores during the first 24 hours after surgery.

**Results:**

A total of 60 patients were included in the study. There was no significant difference in the baseline characteristics between two groups. Numerical rating scale (NRS) pain scores in the postoperative period were lower in the ESPB group but there was no statistical significance. Intraoperative remifentanil consumption was statistically lower in the ESPB group compared to the control group (*P* < 0.01). ESPB also reduced 24-hour opioid consumption (*P* = 0.002). There was no statistical difference in the incidence of adverse events between the two groups.

**Conclusion:**

The use of ESPB in laparoscopic sleeve gastrectomy patients is associated with a significant reduction in intraoperative and 24-hour postoperative opioid consumption.

**Trial registration ID:**

The trial was registered with Clinicaltrials.gov as trial ID-NCT04368195.

## 1. Background

The number of bariatric surgeries has significantly increased recently, and therefore, there is a sharp rise in obese patients undergoing these surgeries.^1^ Obesity poses a significant risk for postoperative complications worldwide. General anesthesia in these patients contributes to the worsening of postoperative respiratory function due to decreased functional residual capacity and the development of pulmonary atelectasis.^2,3^ These patients are also at risk of postoperative respiratory depression because of the use of sedatives and opioids.^3^

In medical practice, a wide range of changes is continually made to improve the standards of perioperative care. Enhanced recovery after surgery (ERAS) has recently gained significant popularity in the perioperative care of patients.^4^ It has been used in a variety of surgeries, including abdominal surgeries.^5^ ERAS is a multifaceted program consisting of evidence-based protocols that are aimed to decrease surgical stress, enhance earlier postoperative recovery, and improve patient outcomes. The components of ERAS broadly consist of preadmission, preoperative, intraoperative, and postoperative phases of patient care. The role of anesthetists in ERAS protocols is to modify perioperative anesthesia care to improve the quality and outcome of patient care.^4^

The use of regional block techniques peri-operatively can help in reducing the requirements of opioids and hence their associated side effects. Regional block techniques, including epidurals, paravertebral blocks, and transverse abdominis plane blocks, are generally considered part of multimodal analgesia in patients undergoing bariatric surgeries like sleeve gastrectomies.^6-8^ Each of these approaches has limitations including longer hospital stays with epidurals, potential risk of pneumothorax with paravertebral, and inadequacy of pain control with transversus abdominis plane (TAP) block.^6,8,9^

Erector spinae plane block (ESPB) is a relatively newer regional block that has the advantage of blocking both visceral and somatic nerve fibers. Additionally, it can be performed using easily identified ultrasound landmarks with a much better safety profile.^10,11^ This block involves injecting local anesthetic in the interfascial plane between the transverse process of the vertebra and erector spinae muscle ultimately resulting in the spread to multiple paravertebral spaces.^8,12^ In one study, Tulgara et al.^13^ demonstrated that single-shot ESPB in laparoscopic cholecystectomy patients resulted in lower pain scores postoperatively and lesser opioid requirements in 24 hours. The analgesic efficacy of ESPB has also been demonstrated in one case series by Chin et al.^10^ So, this study aimed to assess the role of ESPB in reducing postoperative pain scores and opioid consumption in patients undergoing sleeve gastrostomies.

## 2. Methods

This randomized controlled trial was done at a tertiary care hospital in Riyadh, Kingdom of Saudi Arabia. It was approved by the Institutional Ethical Committee with the registration number 20-399-11, and written informed consent was taken from each patient. The trial was registered before patient enrollment at ClinicalTrial.gov (NCT04368195, April 29th, 2020). All the procedures in this trial were conducted in accordance with the Helsinki Declaration-2013.

Patients aged between 18 and 65 years, with American Society of Anesthesiologists (ASA) scores 1–3 who were scheduled to undergo laparoscopic sleeve gastrectomy under general anesthesia were included in our study. Exclusion criteria included patient refusal, later request for removal from the study, inability to consent, contraindications to regional anesthesia, known allergy to local anesthesia, inability to operate patient-controlled analgesia (PCA), psychiatric disorders or use of psychiatric medications, and conversion to open surgery.

The null hypothesis in our study was that there is no difference in postoperative numeric rating scale (NRS) pain scores between ESPB and the control group in patients undergoing laparoscopic sleeve gastrectomy. For sample size calculation, we used the study by Tulgara et al.^13^ which was done on laparoscopic cholecystectomy using a pooled standard deviation of 0.725 and margin of error (d) as 0.6 units on NRS pain score at the significance level (α) of 0.05 and power of 80%. The sample size came out to be 22 in each group and with 10% dropouts/attrition, we took 25 patients in each group.

Upon admission to the ward, a random ID was assigned to each patient after meeting the inclusion criteria. A computer-generated random number table was used. Sealed opaque envelopes were used to determine the study groups once the patient arrived in the operation theater. The envelope was then handed over to the anesthetist responsible for giving anesthesia to the patient. Patients were assigned to either of two groups: Group A (control group) and Group B (ESPB group). Perioperative anesthesia and surgical techniques were standardized in both groups.

On arrival to the room, patients were connected to standard ASA monitoring, including pulse oximetry, ECG, and non-invasive blood pressure. Baseline measurements of heart rate, blood pressure, and oxygen saturation were recorded. After pre-oxygenation for 3 minutes, induction of anesthesia was performed using 1.5–2 mcg/kg intravenous (IV) fentanyl, 2–2.5 mg/kg IV propofol, followed by 0.2 mg/kg IV cisatracurium. Following tracheal intubation, anesthesia was maintained using 0.8–1.2 minimum alveolar concentration (MAC) sevoflurane. IV remifentanil was used at 0.02–1.5 mcg/kg/minute depending on the patient’s hemodynamic parameters. Muscle relaxation was managed using additional boluses of IV cisatracurium as needed.

In the control group, no block was performed. In the ESPB group, the patient was placed in a lateral position after induction of anesthesia. Using all aseptic measures, a linear ultrasound probe (6–10 MHz) was placed in the cranio-caudal direction at the level of the T9 spinous process. The probe was then moved in a parasagittal plane to about 3 cm lateral to the T9 spinous process. The erector spinae muscle was identified as lying superficial to the tip of the T9 transverse process. A 21-gauge 100-mm ultrasound needle was then inserted using an in-plane approach in the cranio-caudal direction. The needle was then progressed into the fascial plane on the deep (anterior) aspect of the erector spinae muscle. The location of the needle was confirmed using hydro-dissection. A total of 15 mL of 0.25% ropivacaine was injected. The block was performed on the other side as well.

At 20–30 minutes before the end of the surgery, 1 g IV paracetamol, 16 mg IV lornoxicam, 8 mg dexamethasone, and 1 mg granisetron were administered to all patients. Muscle relaxation was reversed using IV neostigmine 2.5 mg and glycopyrrolate 0.5 mg. The patient was extubated after achieving a TOF ratio >0.9 and then transferred to the post-anesthesia care unit (PACU).

The numeric rating scale (NRS) was used to evaluate postoperative pain. The NRS is a segmented version of visual analogue scale (VAS) in which a respondent selects 0–10 to reflect the intensity of pain. All patients in the control and the ESPB groups received a standardized postoperative analgesia protocol using IV morphine patient-controlled analgesia (PCA). A total volume of 100 mL with a concentration of 1 mg/mL morphine was used in PCA. There was no basal infusion rate, but 1 mg bolus and a lockout time of 8 minutes were selected. PCA was started in PACU once a patient was able to use it. All patients were given instructions preoperatively about the use of PCA. Rescue analgesia of IV 2 mg morphine was administered if the NRS score of a patient was equal to or more than 4.

The primary outcome measures of the study were NRS scores postoperatively at 15 minutes, 30 minutes, and 1, 2, 6, 12, 18, and 24 hours after operation. Secondary outcome measures of the study included 24-hour opioid consumption, use of rescue analgesia, and the occurrence of adverse events including postoperative nausea and vomiting (PONV) and drowsiness in 24 hours postoperatively. PONV and drowsiness were assessed on a 4-point scale (none, mild, moderate, or severe). All these postoperative observations were recorded in the first 24 hours postoperatively by an assigned nurse who was blinded to the group of study.

SPSS 22.0 statistical package program (SPSS, Chicago, IL, USA) was used for statistical analysis in our study. The normality of all data was first checked. Descriptive statistics were expressed as mean ± standard deviation or median (Q1, Q3). Depending on the normality of the data, continuous variables were compared using an independent t-test or Mann-Whitney U test. Categorical variables were compared using the Chi-square test and Fisher exact test. A P-value of less than 0.05 was considered statistically significant.

## 3. Results

**Figure 1.**
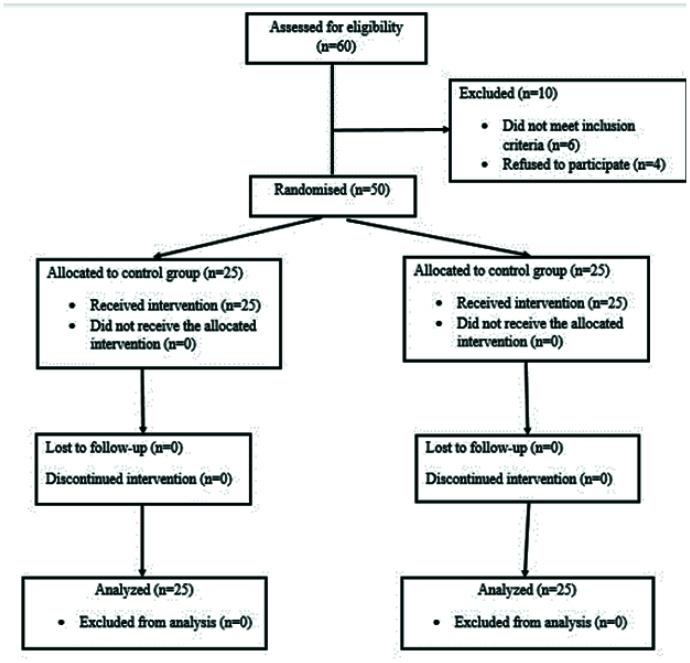
CONSORT flow diagram describing the enrolment of patients in the trail.

The study recruited and followed up participants between 1st April 2020 and 30th December 2022. Sixty patients were considered for eligibility; however, ten were excluded. Of these, six patients did not meet the inclusion criteria, and four denied participating in the study. A total of 50 patients were included in the study and randomized. Twenty-five patients received general anesthesia with no block, while twenty-five patients received general anesthesia with ESPB. The CONSORT diagram is shown in Figure 1.

The median age was 35 (23.5–41.5) years in the control group vs. 37.88 (34–45.5) years in the ESPB group. Nine patients were male in the control and 19 in the ESPB group. The median duration of surgery in the control group was 139 minutes vs. 145 minutes in the ESPB group. There were no significant differences in the demographics of patients between the control and ESPB groups. All outcome variables were not found to have a normal distribution.

Mann-Whitney U test was used to compare the medians of all our outcome variables. Pain scores were found to be lower with ESPB postoperatively, although there was no statistical significance as shown in Table 1. Intraoperative remifentanil consumption was statistically lower in the ESPB group in comparison to the control group, [(Control group median (Q1, Q3) as 950 (900, 1012), ESPB 400 (312, 450)] (P < 0.01). The consumption of opioids at 24 hours was found to be significantly different between the two groups [(control group median (Q1, Q3) as 22 (17, 32), ESPB 13(11, 19)] (P = 0.002), as shown in Table 1. Ten patients in the control group and nine patients in the ESPB group experienced PONV that needed anti-emetics postoperatively in the first 24 hours. None of the patients had drowsiness or shoulder pain postoperatively.

## 4. Discussion

Our study demonstrated that the use of erector spinae block in laparoscopic sleeve gastrectomy reduced 24-hour opioid consumption without increasing any side effects postoperatively. The block also reduced the pain scores postoperatively, but the results did not achieve statistical significance.

The use of regional anesthesia supplements and improves multimodal analgesia after abdominal surgeries and enhances surgical outcomes.^14,15^ ESPB targets the ventral rami, dorsal rami, and rami communicants of the spinal nerves.^16^ Adhikary et al.^17^ in a cadaveric study revealed that ESPB produced an epidural, neural foraminal, and intercostal spread of local anesthesia agents ultimately resulting in a more extensive spread of local anesthetics and hence larger coverage of dermatomal area as compared to transverse abdominal plane (TAP) block.

Our study demonstrated a 40.9% reduction in 24-hour cumulative opioid consumption by using ESPB in laparoscopic sleeve gastrectomy. This reduction is more than what was reported (30%) by Tulgar et al.^13^ in patients undergoing laparoscopic cholecystectomy with ESPB. Our results are also consistent with the study by Abu Elzayed et al.^18^ that included patients undergoing open epigastric hernia repair. They showed that the median pethidine consumption in ESPB was significantly lower than the control group (0 mg vs. 83 mg). In two other studies, ESPB decreased opioid consumption by 47% in patients undergoing laparoscopic cholecystectomy.^19,20^ Intraoperative consumption of intravenous remifentanil in ESBP was also significantly lower by 58% compared to the control group. This finding was consistent with the study by Pecker et al.^20^ that reported 50% lesser intraoperative remifentanil consumption in patients who received ESPB for laparoscopic cholecystectomy. On the other hand, Li et al.^21^ did not find any statistical difference in intraoperative remifentanil consumption with using ESPB in patients undergoing laparoscopic colon cancer surgery. Abu Elzayed et al.^18^ also reported significantly lower intraoperative fentanyl consumption in patients receiving ESPB for open epigastric hernia repair.

Pain scores in our study were not found to be statistically different by using ESPB. This contrasted with the findings by Tulgar et al.^13^ that showed statistically better pain control during the first 3 hours in the ESPB group. Yildiz et al.^19^ also demonstrated significantly lower pain scores in the ESPB group at all time points postoperatively except at 2 hours which was contrary to our results. Abu Elzayed et al.^18^ also showed that pain scores in the ESPB group were significantly lower until 12 hours postoperatively in patients undergoing laparoscopic cholecystectomy. On the other hand, Kwon et al.^22^ did not find any statistically significant different pain scores postoperatively except at 6 hours.

One of the important target outcomes of ERAS is the reduction of associated side effects after surgery. In our study, we had a slightly lesser incidence of PONV (36% vs. 40%) in the ESPB group, although it was not statistically different. This result was consistent with the study by Yildiz et al.^19^ on patients undergoing laparoscopic cholecystectomy that reported the incidence of nausea as 23.5% in the ESPB group in comparison to 35.2% in the control group. Abu Elzayed et al.^18^ also did not report any significant difference in PONV in the two groups, although the overall incidences of PONV were low in their study at 6.67% vs. 10% in the ESPB vs. the control group, respectively.

There are a few limitations in our study. The main limitation is the lack of documentation of the distribution of loss of sensation in the ESPB group. The test for loss of sensation could not be done in our study as we did the block after anesthesia induction to maintain patient blinding in our study. Since we did not perform a block assessment, it is also impossible to rule out a systemic effect of local anesthetics. We did not measure the time to block performance, and the study was not adequately powered to detect block-related complications. Also, this was a single-center study. The number of participants enrolled was small, so the applicability of results may be limited. We did not effectively evaluate any index to distinguish visceral pain from incisional pain, which needs to be confirmed by further studies.

## 5. Conclusion

The use of ESPB in laparoscopic sleeve gastrectomy patients is associated with a significant reduction in intraoperative and 24-hour postoperative opioid consumption by 58% and 41%, respectively. Postoperative pain scores were not statistically better in the ESPB group. We recommend ESPB as a standard regional anesthesia technique for laparoscopic sleeve gastrectomy.

## Acknowledgment

None.

## Conflict of Interest Statement

None.

## Authors’ Contributions

AuH: This author helped with the concept, literature search, conduct of study, data analysis, manuscript writing and editing, and final approval. AA: This author helped with literature search, conduct of study, manuscript editing, and final approval. MY: This author helped with literature search, conduct of study, manuscript editing, and final approval. AS: This author helped with literature search, conduct of study, manuscript editing, and final approval. ANA: This author helped with conduct of study, manuscript editing, and final approval.

## Figures and Tables

**Table 1.** Comparison of postoperative outcome variables between control group and erector spinae plane block (ESPB) group

**Outcome variables**	**Control group (n=25)**	**ESPB group (n=25)**	**P-value**
	**Median (Q1-Q3)**	**Median (Q1-Q3)**	
NRS at 15 minutes postoperatively	4 (2-5)	2 (0-4)	0.572
NRS at 30 minutes postoperatively	3 (1.5-4)	2 (1.5-3)	0.57
NRS at 1 hour postoperatively	2 (0.5-2.5)	2 (1-2)	0.247
NRS at 2 hours postoperatively	2 (2-2.5)	2 (1-2)	0.724
NRS at 6 hours postoperatively	2 (2-2)	2 (0-2)	0.602
NRS at 12 hours postoperatively	1 (1-2)	1 (0-2)	0.572
NRS at 24 hours postoperatively	1 (0-1.5)	1 (0-1)	1.0
Intraoperative remifentanil consumption (mcg)	950 (900-1012)	400 (312-450)	0.00
24 hours opioid consumption (mg)	22 (17-32)	13 (11-19)	0.002

Outcome variables are expressed as median (Q1-Q3), NRS – Numerical rating scale.
